# 嵌合抗原受体T细胞治疗多发性骨髓瘤中国血液临床专家共识（2026年版）

**DOI:** 10.3760/cma.j.cn121090-20260114-00023

**Published:** 2026-04

**Authors:** 中国医师协会血液科医师分会 中华医学会血液学分会

## Abstract

嵌合抗原受体T细胞（chimeric antigen receptor T-cell，CAR-T细胞）免疫疗法在复发难治性多发性骨髓瘤（MM）中显示了较好的疗效和可靠的安全性。自首个共识发表后，CAR-T细胞治疗MM的临床研究和应用在广度和深度上进一步拓展，三款新型B细胞成熟抗原CAR-T细胞被国家药品监督管理局批准应用于临床，新型靶点（如GPRC5D）CAR-T细胞的开发与应用获得了广泛关注，CAR-T细胞治疗后的挽救治疗有新的突破，CAR-T细胞治疗MM的全程管理亦有更新。鉴于此，有必要对CAR-T细胞治疗MM的共识进行修订，以适应MM CAR-T细胞治疗的临床新需求。

## Abstract (en)

Chimeric antigen receptor T-cell（CAR-T）immunotherapy has demonstrated favorable efficacy and reliable safety in relapsed/refractory multiple myeloma（MM）. Since the publication of the first consensus, both the scope and depth of CAR-T clinical research and application in MM have significantly expanded. Notably, three novel B-cell maturation antigen（BCMA）-targeted CAR-T products have been approved by the National Medical Products Administration（NMPA）for clinical use. The development and application of CAR-T targeting novel targets such as GPRC5D have garnered significant attention. Breakthroughs have been achieved in salvage therapies for post-CAR-T relapse, while enhanced understanding has been gained regarding comprehensive management of the CAR-T treatment for MM. In light of these advancements, it is imperative to update the consensus guidelines on CAR-T therapy for MM to address evolving clinical requirements in this rapidly progressing field.

多发性骨髓瘤（MM）是一种恶性血液系统疾病，其特征是骨髓中克隆性浆细胞增殖，导致一系列并发症，包括肾衰竭、骨病变和贫血。在全球范围内，MM约占所有癌症的1％，占血液系统恶性肿瘤的10％左右。依据全球疾病负担（GBD）数据库的数据，2021年，中国预计有17 250例MM新发病例和12 984例死亡病例。每10万人口的年龄标准化发病率、死亡率和患病率分别为0.8、0.6和2.2，从1990年到2021年，年龄标准化的患病率增长幅度明显高于发病率和死亡率的增长幅度，反映了治疗效果的改善和患者生存期的延长，其中新型药物和治疗手段发挥了积极的作用[1]。

嵌合抗原受体T细胞（chimeric antigen receptor T-cells，CAR-T细胞）疗法指通过基因修饰技术将带有特异性抗原识别结构域及T细胞激活信号的遗传物质转入T细胞，使T细胞直接与肿瘤细胞表面的特异性抗原结合而被激活、增殖，从而发挥靶向杀伤肿瘤细胞的作用。2016年，Ali等[2]首次报道抗B细胞成熟抗原（B cell maturation antigen，BCMA）CAR-T细胞应用于复发难治性MM（RRMM），证实了其有效性和安全性。随后国内外开展了大量抗BCMA CAR-T细胞治疗RRMM的临床研究，总体有效率为73％～100％，其中1个月的微小残留病（MRD）转阴率可超过80％。鉴于BCMA CAR-T细胞的疗效及安全性，美国已批准两款以BCMA为靶点的CAR-T细胞，中国已批准三款以BCMA为靶点的CAR-T细胞（西达基奥仑赛、伊基奥仑赛、泽沃基奥仑赛）用于治疗RRMM。除了抗BCMA CAR-T细胞外，GPRC5D、CS1、CD38、CD138、FCRL5等单靶点及BCMA/GPRC5D、BCMA/CD19[3]、BCMA/CD38[4]、BCMA/CD138、BCMA/FCRL5等序贯或双靶点先后进入临床研究。随着CAR-T细胞治疗MM病例的积累，如何延长缓解时间、降低复发率以及复发后的挽救治疗等成为亟需解决的问题。

在降低肿瘤负荷或控制疾病进展的同时，避免增加额外的不良反应或影响CAR-T细胞的应用，在双特异性抗体与CAR-T细胞均可及情况下，应优先选择CAR-T细胞。鉴于CAR-T细胞治疗MM领域的快速发展及对安全性的考虑，规范化管理对未来的临床试验和临床应用非常必要。为此，国内相关专家共同编写了此共识，旨在提高临床CAR-T细胞治疗MM的管理水平。

一、CAR-T细胞治疗前的患者准备

1. 商品化CAR-T细胞的患者准备：

（1）评估适应证：美国已经批准商品化的BCMA CAR-T细胞用于首次复发MM的治疗，但中国目前获批的三款BCMA CAR-T细胞适应证均为三线以上治疗后难治复发患者，包括接受过免疫调节剂、蛋白酶体抑制剂及抗CD38单克隆抗体。

（2）评估患者状态：商品化CAR-T细胞的临床应用参照其说明书，一般对体能状态无特殊要求，对于无更好治疗选择的患者，卡诺夫斯基功能状态（Karnofsky Performance Status，KPS）评分低和美国东部肿瘤协作组（Eastern Cooperative Oncology Group，ECOG）体能状态评分高不是绝对禁忌证。肾功能异常的患者可接受CAR-T细胞治疗，但当肌酐清除率<30 ml/min时需谨慎；CAR-T细胞治疗过程中，心功能不良事件发生率约26％，欧洲血液和骨髓移植学会（EBMT）建议左心室射血分数（LVEF）>40％，但合并心肌淀粉样变性的患者可能表现为射血分数保留型心功能不全，即使LVEF正常，治疗过程中也需要更多关注。体内有外源性植入物（如动/静脉导管、各类假体、静脉滤网等）时，需警惕局部和全身感染。

（3）疾病评估：原发病评估主要包括患者的骨髓瘤负荷、临床分期、髓外病变及器官受累情况等，具体参照《中国多发性骨髓瘤诊治指南（2024年修订）》[5]。关于靶点的评估，BCMA几乎表达于所有异常或正常浆细胞，首次使用CAR-T细胞前一般不常规评估其表达，血清中可溶性BCMA含量与预后相关。对于CAR-T细胞治疗后复发患者，需评估靶抗原表达情况，以明确是否存在抗原逃逸。

2. CAR-T细胞临床试验的患者准备：目前及未来开展的CAR-T细胞治疗相关临床试验主要包括：①新型靶点的临床验证；②BCMA、GPRC5D等成熟靶点的优化；③双靶点或多靶点CAR-T细胞的临床验证；④CAR-T细胞与单克隆抗体、小分子药物、免疫检查点抑制剂、免疫调节剂、造血干细胞移植的联合；⑤CAR-T细胞治疗线数前移，包括首次复发及高危患者的一线治疗；⑥髓外病变、CAR-T细胞治疗后的维持和挽救治疗及并发症的管理等。主要临床试验见表1。

**表1:** 2022年1月至2025年6月CAR-T细胞治疗多发性骨髓瘤的代表性临床研究

作者	例数	产品名称	共刺激分子	CAR-T细胞剂量	CRS发生率（%）	≥3级CRS发生率（%）	NTX发生率（%）	≥3级NTX发生率（%）	ORR（%）	≥CR率（%）	中位随访时间（月）	中位EFS期（月）	中位PFS期（月）
Qu等[6]	31	C-CAR088	4-1BB	（1、3、6）×10^6^/kg	93.5	9.7	3.2	0	96.4	57.1	9.4	N/A	N/A
Mailankody等[7]	43	ALLO-715	4-1BB	（40～480）×10^6^	55.8	2.3	14	0	55.8	25	10.2	N/A	N/A
Rodriguez-Otero等[8]	225	Ide-cel	4-1BB	（175～529）×10^6^	88	5	15	3	71	39	18.6	N/A	13.3
Lin等[9]	62	Ide-cel	4-1BB	（150～450）×10^6^	75.8	6.5	37.1	1.6	75.8	38.7	18.1	N/A	8.8
Qiang等[10]	22	GC012F	4-1BB	（1～3）×10^6^/kg	27	0	0	0	100	100	16	N/A	未达到
Zhou等[11]	21	anti-BCMA/GPRC5D	4-1BB	（0.5～4.0）×10^6^/kg	71	0	4.8	0	86	62	5.8	N/A	未达到
Shi等[12]	50	BC19	4-1BB	1×10^6^/kg	92	8	4	0	92	60	N/A	N/A	19.7
San-Miguel等[13]	176	Cilta-cel	4-1BB	0.75×10^6^/kg	76.1	1.1	20.5	2.8	84.6	73.1	15.9	N/A	未达到
Ravi等[14]	65	BMS-986354	4-1BB	（20、40、80）×10^6^	82	2	8	2	95	46	10.2（≥PR患者中位随访时间）	N/A	12.3
Jagannath等[15]	97	CARTITUDE-1	4-1BB	0.75×10^6^/kg	95	4	21	9	97	67	33.4	N/A	34.9
Jurgens等[16]	17	MCARH109	4-1BB	（25、50、150、450）×10^6^	88	6	18	18	71	41	37	N/A	N/A

**注** CAR-T细胞：嵌合抗原受体T细胞；CRS：细胞因子释放综合征；NTX：神经毒性；ORR：总缓解率；CR：完全缓解；EFS：无事件生存；PFS：无进展生存；N/A：无数据；PR：部分缓解

（1）评估适应证及患者状态：按照相应临床研究的入组与排除要求。一般要求：①KPS评分≥50％或ECOG评分≤2分；②具有良好的心、肺、肝功能，LVEF≥50％；ALT、AST<正常值范围上限的3倍、总胆红素<34.2 µmol/L；室内空气下患者的血氧饱和度≥92％；③无活动性感染；④预计生存期>12周。同时应排除：①合并心、肺、脑等重要脏器明显功能障碍的患者；②怀孕、哺乳期或半年内有妊娠计划的女性患者；③伴有传染性疾病（如活动性乙型病毒性肝炎、丙型病毒性肝炎或活动性结核等）、生命体征不正常及不能配合检查者；④有精神或心理疾病不能配合治疗及疗效评估者；⑤对CAR-T细胞产品中任何一种有效成分有过敏史者。

（2）疾病评估：原发病评估主要包括患者骨髓瘤负荷、临床分期、髓外病变及器官受累等，具体评估参照《中国多发性骨髓瘤诊治指南（2024年修订）》[5]。其他靶点（GPRC5D、CS1、CD38、FCRL5等）建议评估其表达情况。

二、CAR-T细胞的制备

1. 采集CAR-T细胞所需淋巴细胞前的药物洗脱期：MM患者前期接受的治疗药物可能影响CAR-T细胞的活性，一般建议采集细胞前8周内未接受抗T细胞单克隆抗体、供者淋巴细胞输注及中枢神经系统放疗；2周内未接受过联合化疗等治疗[17]–[18]。对于半衰期较短者，如来那度胺、低剂量地塞米松及维奈克拉，只要洗脱5个药物半衰期即可（可短于14 d）。鉴于前期治疗线数及部分药物可能会影响CAR-T细胞活性，对于使用双特异性抗体治疗的患者，可考虑提前采集淋巴细胞备用。对于既往接受过含苯达莫司汀或氟达拉滨治疗的患者，自体CAR-T细胞制备失败的可能性增大。

2. 采集CAR-T细胞制备所需淋巴细胞：一般要求患者/供者HGB>80 g/L，PLT>50×10^9^/L（若因疾病进展导致贫血或血小板减少，评估患者的获益与风险，HGB和PLT基线水平不是淋巴细胞采集的绝对限制因素），外周血中淋巴细胞绝对计数>0.5×10^9^/L或CD3^+^淋巴细胞绝对计数>0.15×10^9^/L，淋巴细胞采集量一般为（60～600）×10^6^/kg[5]。

3. CAR-T细胞的制备：采集成功的淋巴细胞将通过冷链由专人运输至生产车间，不同产品制备流程不同，商品化CAR-T细胞有更严格的病原微生物、细胞扩增数量等检测要求，制备所需时间较长，有制备失败风险。制备成功达到放行标准后会通知临床以便清除淋巴细胞。

三、CAR-T细胞回输前的管理

1. 桥接治疗的适应证：存在以下情况时可考虑桥接治疗：①肿瘤负荷重或伴巨大髓外病灶；②CAR-T细胞制备期间疾病进展迅速，可能影响CAR-T细胞治疗的疗效或增加不良反应；③各种原因导致无法按时输注CAR-T细胞。

2. 桥接治疗的方案：目前尚无统一的桥接治疗方案，已发表的数据显示，结合患者前期治疗的暴露及敏感情况，双特异性抗体、抗CD38单克隆抗体、塞利尼索、免疫调节剂、蛋白酶体抑制剂、放疗均可作为桥接治疗方案，使用单克隆抗体或双特异性抗体桥接治疗时避免使用同靶点或可能下调靶抗原表达的药物。传统联合化疗因不良反应较大，不作为优先考虑。

3. 其他：对于缓解程度好或肿瘤负荷较低患者可不桥接，但需密切监测疾病状态，避免感染，等待CAR-T细胞制备成功。

4. 淋巴细胞清除：淋巴细胞清除的目的是抑制体内淋巴细胞功能，使制备好的CAR-T细胞在接触肿瘤抗原发生扩增前不被体内免疫系统清除。常用的淋巴细胞清除方案包括环磷酰胺联合氟达拉滨或环磷酰胺单药等，环磷酰胺300 mg/m^2^×3 d，氟达拉滨30 mg/m^2^×3 d。肾功能不全时的剂量调整参阅环磷酰胺和氟达拉滨的处方信息。

四、CAR-T细胞输注的管理

1. CAR-T细胞输注前：感染的筛查、预防和管理参见毒性管理中感染部分。既往伴中枢神经系统疾病或并发症的患者发生神经系统不良事件的风险增高，建议待疾病控制后再行CAR-T细胞输注（原发病中枢神经系统受累除外），同时可口服左乙拉西坦等药物预防癫痫发生。

2. CAR-T细胞输注期间：预处理化疗结束后2 d输注CAR-T细胞，最长不宜超过7 d。商品化CAR-T细胞的输注剂量参照其说明书和前期临床研究，不同的CAR-T细胞产品输注剂量差异很大[3]–[4],[17]–[25]；鉴于临床研究的CAR-T细胞来源于不同公司或实验室，具体剂量要依据各产品前期预实验的推荐剂量。CAR-T细胞输注前开始进行生命体征监测[26]，不推荐CAR-T细胞输注前予糖皮质激素预防过敏反应。

3. CAR-T细胞输注后：持续性监测生命体征至细胞因子释放综合征（CRS）症状消失，尤其是发生2级或以上CRS时，监护直至CRS分级降至≤1级或根据患者的一般情况而定[26]–[28]。CAR-T细胞输注后患者应住院观察，建议至少在医院密切监测7～14 d[27]–[28]。发生CRS时每天进行体格检查并监测血常规、生化指标和凝血功能、血气分析、血清C反应蛋白、铁蛋白、IL-6等，常规监护下至少每4小时评估1次生命体征[3],[27]–[29]。

五、CAR-T细胞治疗相关毒副作用管理

1. CRS：CRS的评估建议采用美国移植和细胞治疗学会（ASTCT）评估标准（表2）[30]。MM CAR-T细胞治疗后CRS的发生率可高达90％，其中3～5级CRS发生率为5％左右[3]–[4],[17]–[25]。CRS的临床表现多样，与受累的组织器官有关，其中发热是常见的首发表现。如果3周内出现以下4种症状或体征之一[26]，即应考虑CRS：①发热，体温≥38 °C；②低血压，收缩压<90 mmHg（1 mmHg＝0.133 kPa）；③动脉血氧饱和度<90％；④出现器官毒性。鉴于以上均为非特异性临床表现，诊断CRS必须排除其他并发症，包括感染、肿瘤溶解综合征及过敏反应等[30]。CRS也可迟至4周后发生，对有迟发CRS表现者，仍要警惕。托珠单抗和糖皮质激素是治疗严重或危及生命CRS的主要药物，可根据不同CAR-T产品的安全性推荐，适时应用托珠单抗和（或）糖皮质激素（包括一线应用），在症状消失之前，应密切监测CRS患者的心脏和其他器官功能，同时考虑重症监护和积极支持治疗。CRS分级管理见表3，具体参照CAR-T细胞治疗相关毒性评估和管理建议[26],[30]。伴有髓外病灶的患者需要关注局部CRS反应，一般晚于全身CRS反应，可表现为病灶局部的红、肿、热、痛等炎性反应；对于特殊部位的髓外病变（脊髓腔、胸膜腔、颅内），因治疗期间局部病灶可能出现假性进展或炎症反应而导致局部受压或积液，需关注并积极处理。

**表2:** 细胞因子释放综合征（CRS）分级评估指标及依据

CRS分级	评估指标及依据
1级	发热（体温>38 °C，伴或不伴其他体征），且排除其他发热原因
2级	发热伴低血压（不需应用升压药）和（或）低血氧（需要低流量吸氧）
3级	发热伴低血压（需要一种或不需应用升压药）和（或）低血氧（需要高流量鼻导管、面罩吸氧，但不需要借助机械通气）
4级	发热伴低血压（需要多种升压药，但不包括血管加压素）和（或）低血氧（需正压通气，包括持续气道正压通气、双水平气道正压通气、气管插管和机械通气）

**表3:** 细胞因子释放综合征（CRS）分级管理建议

CRS分级	治疗措施
1级	密切监护，支持治疗，评估感染，检测体液平衡，按需使用解热镇痛药物
2级	密切监护，支持治疗，检测心脏和其他脏器功能，可使用托珠单抗和（或）糖皮质激素
3级	密切监护，支持治疗，使用托珠单抗和（或）糖皮质激素
4级	密切监护，支持治疗，使用托珠单抗和（或）糖皮质激素

**注** 托珠单抗用法为8 mg/kg（单次剂量不超过800 mg），静脉滴注时间大于1 h，控制不佳者可8 h后再次使用，24 h内不超过4次，总次数不得超过4次

2. 噬血细胞综合征（hemophagocytic lymphohistiocytosis，HLH）：对于一线治疗干预后12 h内发热、终末器官毒性（如缺氧、低血压）等未改善者，需警惕并发HLH/巨噬细胞活化综合征（MAS），为了更好地管理HLH/MAS，ASTCT将其统一定义为免疫效应细胞相关的噬血细胞性淋巴组织细胞增生症样综合征（IEC-HS），发生率低于8％，但死亡率极高。由于其与CRS的体征和症状存在重叠，诊断颇具挑战性，建议在临床高度怀疑但尚未完全符合IEC-HS诊断标准时尽早进行干预，建议先阻断IL-6并使用皮质类固醇，随后追加细胞因子阻断剂［如阿那白滞素（IL-1受体拮抗剂）］及靶向治疗药物（如芦可替尼）。依帕伐单抗已获美国FDA批准用于原发性HLH，最近有研究报道其用于治疗B细胞急性淋巴细胞白血病患儿的IEC-HS[31]。此外，如果有CAR-T细胞扩增或持续存在的证据，应考虑使用细胞毒性药物，如环磷酰胺或抗胸腺细胞球蛋白。

3. 神经毒性：MM患者BCMA CAR-T细胞治疗后神经毒性的发生率为10％～42％，其中3级及以上的发生率为1％～3％[30]。GPRC5D CAR-T细胞治疗后神经毒性的发生率为0～9.1％，其中3级及以上的发生率为0～3.0％[32]–[34]。神经毒性表现多样，评估项目除意识、语言、书写外，脑电图检查、眼底检查评估视乳头水肿、脑部影像学检查、腰椎穿刺术测定颅内压等根据需要及时评估[26],[30]；免疫效应细胞相关神经毒性综合征（ICANS）根据ICE积分、意识状态、颅内压升高/脑水肿、癫痫或运动能力下降情况分为1～4级[32]，ICANS的分级管理见表4。此外，CAR-T细胞治疗后并发可逆性后部脑病综合征（PRES）的罕见病例亦有报道。除急性神经毒性外，临床需高度警惕延迟性及特殊类型神经毒性（通常发生在回输后2周至数月），BCMA CAR-T细胞可出现帕金森样症状（面部表情减少、书写过小症、运动迟缓及静止性震颤）和面神经麻痹症状[14]，GPRC5D CAR-T细胞可出现迟发性小脑毒性，表现为头晕、步态不稳、眼球震颤及共济失调，严重者可出现构音障碍[31]。对于上述延迟性毒性，建议在长期随访中加强神经系统查体，必要时完善脑部MRI及脑脊液检查。可参照国际合作共济失调量表（ICARS）或共济失调评估与评定量表（SARA）评估严重程度，评估为轻度时无需特殊处理或仅进行康复训练；评估为中、重度时，除进行康复训练外，需综合治疗。

**表4:** 免疫效应细胞相关神经毒性综合征（ICANS）的分级管理

ICANS分级	未并发CRS	并发CRS的附加治疗
1级	• 支持治疗	• 托珠单抗8 mg/kg，静脉滴注
2级	• 支持治疗 • 地塞米松10 mg，静脉注射（或静脉滴注），每6小时1次；或甲泼尼龙1 mg/kg，静脉滴注，每12小时1次	• 托珠单抗8 mg/kg，静脉滴注 • 若并发≥2级CRS，考虑将患者转至ICU
3级	• 建议转移至ICU • 支持治疗 • 地塞米松10 mg，静脉注射（或静脉滴注），每6小时1次；或甲泼尼龙1 mg/kg，静脉滴注，每12小时1次 • 若ICANS持续≥3级，每2～3天重复进行神经影像学（CT或MRI）检查	• 托珠单抗8 mg/kg，静脉滴注
4级	• 支持治疗 • ICU监护，建议机械通气 • 大剂量激素（甲泼尼龙1 g/d，静脉滴注） • 若ICANS持续≥3级，每2～3天重复进行神经影像学（CT或MRI）检查 • 参照指南治疗惊厥性癫痫持续状态患者	• 支持治疗 • ICU监护，建议机械通气 • 大剂量激素（甲泼尼龙1 g/d，静脉滴注） • 若ICANS持续≥3级，每2～3天重复进行神经影像学（CT或MRI）检查 • 参照指南治疗惊厥性癫痫持续状态患者

**注** CRS：细胞因子释放综合征；ICU：重症监护病房

4. 感染：感染是CAR-T细胞治疗中的重要并发症[30]，常与CRS同时发生，病原菌包括细菌、病毒、真菌、支原体及其他。Wang等[35]的研究显示，BCMA CAR-T细胞治疗MM时，57.4％的患者并发感染，以下呼吸道为主（53％），其次为上呼吸道感染（21％）。病原体依次为细菌（57％）、病毒（18％）、真菌（7％）、支原体（2％）及其他（16％）。CAR-T细胞治疗前需全面筛查（包括但不限于人类免疫缺陷病毒、乙型肝炎病毒、丙型肝炎病毒、巨细胞病毒、结核分枝杆菌等）和控制活动性感染；预处理开始时予阿昔洛韦或伐昔洛韦，用药时间根据免疫功能恢复情况而定。输注CAR-T细胞至粒细胞恢复前予诺氟沙星、三唑类抗真菌药及复方磺胺甲噁唑等预防感染，其中卡氏肺囊虫感染预防至少3个月。感染治疗可参考《中国中性粒细胞缺乏伴发热患者抗菌药物临床应用指南（2016年版）》[36]进行管理；发生3～4度粒细胞减少时，有条件者可隔离保护，同时应用粒细胞集落刺激因子（G-CSF）[26]–[28],[36]。已发表的数据和临床实践显示，CAR-T细胞治疗期间并发新型冠状病毒或其他病毒感染时会增加患者的整体救治风险，建议接受浆细胞靶向CAR-T细胞治疗的患者持续密切监测新型冠状病毒和其他病毒感染，优化感染预防策略，并在必要时给予积极治疗。

5. B细胞缺乏和低免疫球蛋白血症：低免疫球蛋白血症是MM患者CAR-T细胞治疗后常见的并发症，BCMA单靶点治疗时几乎所有患者发生低免疫球蛋白血症和B细胞缺乏。B细胞恢复至基线的中位时间为79 d，1年时IgG、IgA及IgM恢复至正常水平的比例分别为53.33％、23.81％及73.08％。BCMA/CD19双靶点治疗时，低免疫球蛋白血症和B细胞缺乏的发生率为100％，其中B细胞一般于CAR-T细胞输注后2个月逐渐恢复，IgM于3个月后开始恢复，而低IgA和IgG持续时间甚至超过1年[3],[37]，可能增加感染机会。CAR-T细胞治疗后3个月内应每月监测患者血清IgG水平，血清IgG<4 g/L者或血清IgG 4～6 g/L且并发感染者，应用丙种球蛋白替代治疗；血清IgG>6 g/L且并发感染者，建议进一步评估各型免疫球蛋白水平（IgG、IgA及IgM）和B细胞数量。

6. 免疫效应细胞相关血液毒性（ICAHT）：MM患者接受CAR-T细胞治疗较其他B细胞肿瘤有更严重的ICAHT，KarMMa研究显示，3～4级粒细胞、血小板减少和贫血的发生率分别为89％、52％、60％[18]，应积极对症和支持治疗，CAR-HEMATOTOX积分系统可作为预测模型指导管理[38]。

7. 其他：其他治疗相关不良反应包括凝血功能异常、肿瘤溶解综合征、免疫效应细胞相关小肠结肠炎等，具体可参照NCCN免疫治疗相关毒性管理[39]和CAR-T细胞治疗毒性评估和管理[26]。发生CRS的过程中常伴重要脏器功能受累和损伤，包括心脏、消化道、肝脏、肌肉、胰腺等，其中以心脏受损和消化道出血风险最大[39]。具体可参照NCCN免疫治疗相关毒性管理[39]。随着新型靶点的应用，需关注GPRC5D CAR-T细胞治疗相关的靶向非肿瘤毒性（on-target off-tumor toxicities），主要累及表达GPRC5D的角化组织及黏膜。患者可出现皮肤（皮疹、红斑、脱屑及瘙痒）及指甲毒性，约20％患者并发指甲脱落，无需特殊处理，可再生。也可出现味觉障碍（味觉减退、丧失或进食困难），以对症支持治疗为主。二次肿瘤是另一个需要关注的不良事件，截至目前，国内研究未见相关报道，但在KarMMa-3和CARTITUDE-4研究中均有报道[13]。

六、疗效评估

CAR-T细胞治疗后采用国际骨髓瘤工作组（IMWG）和《中国多发性骨髓瘤诊治指南（2024年修订）》[5]进行疗效评估。鉴于CAR-T细胞治疗MM时，大部分治疗有效患者1个月内获得骨髓MRD转阴（流式细胞术）[3],[18],[40]，但免疫固定电泳转阴或轻链比例正常需要更长时间，部分患者甚至在6个月时才能达到最佳疗效[3]。推荐在CAR-T细胞输注后第14、28天评估疗效，并在半年内的每个月进行疗效评估。伴髓外病变者，建议在CAR-T细胞治疗1个月后评估髓外病变，MRI、CT或X线检查均可作为评估手段，3个月后可考虑PET-CT评估。

七、CAR-T细胞治疗后维持治疗

目前，CAR-T细胞治疗后是应否维持治疗或如何维持治疗无基于循证医学的证据。维持治疗是CAR-T细胞未来重点探索的方向之一，包括单克隆抗体或双特异性抗体、蛋白酶体抑制剂、免疫调节剂、造血干细胞移植、纠正免疫微环境等，其中CAR-T细胞与自体造血干细胞移植联合以及年轻患者CAR-T细胞治疗达完全缓解后序贯异基因造血干细胞移植已在开展临床研究；异基因造血干细胞移植后患者可能伴有持续免疫功能低下，参照异基因造血干细胞移植常规监测和管理方案[41]。

八、复发或进展后挽救性治疗

CAR-T细胞治疗后复发的治疗可参照《中国多发性骨髓瘤诊治指南（2024年修订）》[5]中建议的治疗，包括临床试验、既往敏感的药物和未使用的新药或新的联合治疗方案。已发表的数据显示，靶向免疫治疗为优先挽救治疗手段，其中以CAR-T细胞的治疗反应率最高，但需要考虑以下因素：①全面评估复发的原因和靶抗原表达情况；②不推荐再次应用原CAR-T细胞挽救治疗；③靶抗原表达降低或阴性复发患者，只能选择可检测到的其他靶抗原；④CAR-T细胞治疗后3个月内复发患者建议优先选择单克隆抗体、双特异性抗体或与其他新型药物，联合使用可能有更好的疗效和较持久的缓解期；⑤对于CAR-T细胞在体内持续时间短或扩增差的患者，首先评估患者T细胞和CAR-T细胞功能或是否存在影响T细胞活性的因素[39]。

九、随访

随访应包括以下三方面内容：原发病持续缓解情况、远期不良反应及感染的防治。CAR-T细胞治疗后14 d和28 d评估，半年内每月评估1次，6～12个月每2个月评估1次，主要评估疾病的缓解状况和不良反应；第二年每3个月进行1次全面评估，第三年（及以后），每6个月或根据临床情况进行全面评估。评估的指标包括M蛋白定量、血清蛋白电泳、免疫固定电泳及游离轻链检测、骨髓细胞学及MRD；有髓外病变者还需完善相关影像学检查，包括皮肤、软组织、淋巴结、肝脏、脾脏及中枢神经系统MRI，必要时行PET-CT检查。对于考虑疾病进展的患者应立即予以评估。感染防治参见“五、CAR-T细胞治疗相关毒副作用管理”中感染防治和低免疫球蛋白血症管理。此外，所有接受以病毒为载体制备的CAR-T细胞治疗患者，均需监测远期生物安全性。

## References

[1] Dou X, Duan G, Zhong Y (2025). The burden of multiple myeloma in China: trends from 1990 to 2021 and forecasts for 2050[J]. Cancer Lett.

[2] Ali SA, Shi V, Maric I (2016). T cells expressing an anti-B-cell maturation antigen chimeric antigen receptor cause remissions of multiple myeloma[J]. Blood.

[3] Yan Z, Cao J, Cheng H (2019). A combination of humanised anti-CD19 and anti-BCMA CAR T cells in patients with relapsed or refractory multiple myeloma: a single-arm, phase 2 trial[J]. Lancet Haematol.

[4] Mei H, Li C, Jiang H (2021). A bispecific CAR-T cell therapy targeting BCMA and CD38 in relapsed or refractory multiple myeloma[J]. J Hematol Oncol.

[5] 中国医师协会血液科医师分会, 中华医学会血液学分会 (2024). 中国多发性骨髓瘤诊治指南(2024年修订)[J]. 中华内科杂志.

[6] Qu X, An G, Sui W (2022). Phase 1 study of C-CAR088, a novel humanized anti-BCMA CAR T-cell therapy in relapsed/refractory multiple myeloma[J]. J Immunother Cancer.

[7] Mailankody S, Matous JV, Chhabra S (2023). Allogeneic BCMA-targeting CAR T cells in relapsed/refractory multiple myeloma: phase 1 UNIVERSAL trial interim results[J]. Nat Med.

[8] Rodriguez-Otero P, Ailawadhi S, Arnulf B (2023). Ide-cel or standard regimens in relapsed and refractory multiple myeloma[J]. N Engl J Med.

[9] Lin Y, Raje NS, Berdeja JG (2023). Idecabtagene vicleucel for relapsed and refractory multiple myeloma: post hoc 18-month follow-up of a phase 1 trial[J]. Nat Med.

[10] Qiang W, Lu J, Jia Y (2024). B-Cell maturation antigen/CD19 dual-targeting immunotherapy in newly diagnosed multiple myeloma[J]. JAMA Oncol.

[11] Zhou D, Sun Q, Xia J (2024). Anti-BCMA/GPRC5D bispecific CAR T cells in patients with relapsed or refractory multiple myeloma: a single-arm, single-centre, phase 1 trial[J]. Lancet Haematol.

[12] Shi M, Wang J, Huang H (2024). Bispecific CAR T cell therapy targeting BCMA and CD19 in relapsed/refractory multiple myeloma: a phase I/II trial[J]. Nat Commun.

[13] San-Miguel J, Dhakal B, Yong K (2023). Cilta-cel or standard care in lenalidomide-refractory multiple myeloma[J]. N Engl J Med.

[14] Ravi G, Richard S, Kumar S (2025). Phase 1 clinical trial of B-cell maturation antigen (BCMA) NEX-T® chimeric antigen receptor (CAR) T cell therapy CC-98633/BMS-986354 in participants with triple-class exposed multiple myeloma[J]. Leukemia.

[15] Jagannath S, Jackson CC, Schecter JM (2024). Cilta-cel, a BCMA-targeting CAR-T therapy for patients with multiple myeloma[J]. Expert Opin Biol Ther.

[16] Jurgens EM, Firestone RS, Chaudhari J (2025). Phase I Trial of MCARH109, a G protein-coupled receptor class C group 5 member D (GPRC5D)-targeted chimeric antigen receptor T-cell therapy for multiple myeloma: an updated analysis[J]. J Clin Oncol.

[17] Brudno JN, Maric I, Hartman SD (2018). T cells genetically modified to express an anti-B-cell maturation antigen chimeric antigen receptor cause remissions of poor-prognosis relapsed multiple myeloma[J]. J Clin Oncol.

[18] Munshi NC, Anderson LD, Shah N (2021). Idecabtagene vicleucel in relapsed and refractory multiple myeloma[J]. N Engl J Med.

[19] Zah E, Nam E, Bhuvan V (2020). Systematically optimized BCMA/CS1 bispecific CAR-T cells robustly control heterogeneous multiple myeloma[J]. Nat Commun.

[20] Berdeja JG, Madduri D, Usmani SZ (2021). Ciltacabtagene autoleucel, a B-cell maturation antigen-directed chimeric antigen receptor T-cell therapy in patients with relapsed or refractory multiple myeloma (CARTITUDE-1): a phase 1b/2 open-label study[J]. Lancet.

[21] Li CR, Wang QX, Zhu H (2018). T cells expressing anti B-cell maturation antigen chimeric antigen receptors for plasma cell malignancies[J]. Blood.

[22] Zhao WH, Liu J, Wang BY (2018). Updated analysis of a phase 1, open-label study of LCAR-B38M, a chimeric antigen receptor T cell therapy directed against B-cell maturation antigen, in patients with relapsed/refractory multiple myeloma[J]. Blood.

[23] Li CR, Wang J, Wang D (2019). Efficacy and safety of fully human BCMA targeting CAR T cell therapy in relapsed/refractory multiple myeloma[J]. Blood.

[24] Weijun Fu, Juan Du, Hua Jiang (2019). Efficacy and safety of CAR-T therapy with safety switch targeting BCMA for patients with relapsed/refractory multiple myeloma in a phase 1 clinical study[J]. Blood.

[25] Jin Jie, Siguo Hao, Songfu Jiang (2019). Phase 1 trial of the safety and efficacy of fully human anti-BCMA CAR T cells in relapsed/refractory multiple myeloma[J]. Blood.

[26] Neelapu SS, Tummala S, Kebriaei P (2018). Chimeric antigen receptor T-cell therapy - assessment and management of toxicities[J]. Nat Rev Clin Oncol.

[27] Kansagra AJ, Frey NV, Bar M (2019). Clinical utilization of chimeric antigen receptor T-cells (CAR-T) in B-cell acute lymphoblastic leukemia (ALL)-an expert opinion from the European Society for Blood and Marrow Transplantation (EBMT) and the American Society for Blood and Marrow Transplantation (ASBMT)[J]. Bone Marrow Transplant.

[28] Yakoub-Agha I, Chabannon C, Bader P (2020). Management of adults and children undergoing chimeric antigen receptor T-cell therapy: best practice recommendations of the European Society for Blood and Marrow Transplantation (EBMT) and the Joint Accreditation Committee of ISCT and EBMT (JACIE)[J]. Haematologica.

[29] Yan Z, Zhang H, Cao J (2021). Characteristics and risk factors of cytokine release syndrome in chimeric antigen receptor T cell treatment[J]. Front Immunol.

[30] Lee DW, Santomasso BD, Locke FL (2019). ASTCT consensus grading for cytokine release syndrome and neurologic toxicity associated with immune effector cells[J]. Biol Blood Marrow Transplant.

[31] Scala JJ, Eckrich MJ, Lipak K (2024). Treatment strategies for progressive immune effector cell-associated hemophagocytic lymphohistiocytosis-like syndrome: case series[J]. Haematologica.

[32] Mailankody S, Devlin SM, Landa J (2022). GPRC5D-targeted CAR T cells for myeloma[J]. N Engl J Med.

[33] Zhang M, Wei G, Zhou L (2023). GPRC5D CAR T cells (OriCAR-017) in patients with relapsed or refractory multiple myeloma (POLARIS): a first-in-human, single-centre, single-arm, phase 1 trial[J]. Lancet Haematol.

[34] Xia J, Li H, Yan Z (2023). Anti-G protein-coupled receptor, class C group 5 member D chimeric antigen receptor T cells in patients with relapsed or refractory multiple myeloma: a single-arm, phase Ⅱ trial[J]. J Clin Oncol.

[35] Wang Y, Li C, Xia J (2021). Humoral immune reconstitution after anti-BCMA CAR T-cell therapy in relapsed/refractory multiple myeloma[J]. Blood Adv.

[36] 中华医学会血液学分会, 中国医师协会血液科医师分会 (2016). 中国中性粒细胞缺乏伴发热患者抗菌药物临床应用指南(2016年版)[J]. 中华血液学杂志.

[37] 葛 娇, 赵 婷婷, 万 重阳 (2021). 单纯输注抗BCMA与抗BCMA输注联合抗CD19嵌合抗原受体T细胞治疗复发/难治性多发性骨髓瘤免疫重建的对比[J]. 中华血液学杂志.

[38] Rejeski K, Hansen DK, Bansal R (2023). The CAR-HEMATOTOX score as a prognostic model of toxicity and response in patients receiving BCMA-directed CAR-T for relapsed/refractory multiple myeloma[J]. J Hematol Oncol.

[39] Thompson JA, Schneider BJ, Brahmer J (2020). NCCN Guidelines Insights: management of immunotherapy-related toxicities, Version 1.2020[J]. J Natl Compr Canc Netw.

[40] Raje N, Berdeja J, Lin Y (2019). Anti-BCMA CAR T-cell therapy bb2121 in relapsed or refractory multiple myeloma[J]. N Engl J Med.

[41] Wang Y, Chang YJ, Chen J (2024). Consensus on the monitoring, treatment, and prevention of leukaemia relapse after allogeneic haematopoietic stem cell transplantation in China: 2024 update[J]. Cancer Lett.

